# Morphological priming during language switching: an ERP study

**DOI:** 10.3389/fnhum.2014.00995

**Published:** 2014-12-12

**Authors:** Saskia E. Lensink, Rinus G. Verdonschot, Niels O. Schiller

**Affiliations:** ^1^Faculty of Humanities, Leiden University Centre for Linguistics, Leiden UniversityLeiden, Netherlands; ^2^Leiden Institute for Brain and Cognition, Leiden UniversityLeiden, Netherlands; ^3^Graduate School of Languages and Cultures, Nagoya UniversityNagoya, Japan

**Keywords:** morphological priming, compounds, bilingual language processing, language switch, ERP

## Abstract

Bilingual language control (BLC) is a much-debated issue in recent literature. Some models assume BLC is achieved by various types of inhibition of the non-target language, whereas other models do not assume any inhibitory mechanisms. In an event-related potential (ERP) study involving a long-lag morphological priming paradigm, participants were required to name pictures and read aloud words in both their L1 (Dutch) and L2 (English). Switch blocks contained intervening L1 items between L2 primes and targets, whereas non-switch blocks contained only L2 stimuli. In non-switch blocks, target picture names that were morphologically related to the primes were named faster than unrelated control items. In switch blocks, faster response latencies were recorded for morphologically related targets as well, demonstrating the existence of morphological priming in the L2. However, only in non-switch blocks, ERP data showed a reduced N400 trend, possibly suggesting that participants made use of a post-lexical checking mechanism during the switch block.

## INTRODUCTION

It is not clear how morphologically complex words are represented and processed in non-native speakers. This study aims to shed more light on this issue by means of an overt speech production experiment where both behavioral and event-related potential (ERP) data were collected. Participants were presented with a morphological priming task where Dutch speakers with English as their L2 were required to read aloud words and name pictures in both English and Dutch. The results of this study not only provide more insights into the issue of how morphologically complex words of the L2 are represented in the brain, but also inform theories concerning bilingual language control.

Bilingual language control has been a much-debated issue in the literature over the past few years (Green, 1998, 2011; Christoffels et al., 2007; Abutalebi and Green, 2008; Colzato et al., 2008; Verdonschot et al., 2012; Bobb and Wodniecka, 2013). People who are fluent in more than one language are quite capable of keeping their languages apart. This process seems to be effortless and usually is without intrusions from one language into the other (Poulisse, 1999). This is particularly striking considering the evidence suggesting that both languages of bilinguals are active, even when only one is being used (Green, 1986; Kroll et al., 2006; Van Heuven et al., 2008).

It is generally assumed that in language production, lexical items compete for selection (Levelt et al., 1999; Bloem and La Heij, 2003; but see Mahon et al., 2007), and that the item with the highest level of activation wins. In bilingual language production this would potentially pose problems, as the same concept would activate two lexical representations in both language A and language B. There are several models accounting for how bilinguals manage selecting the target language. One type of model assumes an active or reactive inhibition of the non-target language – the Inhibitory Control or IC models (Green, 1998, 2011). Conversely, activation levels of the target language could be raised, so that no inhibitory processes need to be posited to achieve selection of the correct language – the non-Inhibitory Control or non-IC models (Costa et al., 1999; Costa and Santesteban, 2004). A third possibility concerns a hybrid model, where both inhibition of the non-target language and raising activation levels of the target language occur, with context influencing which process is employed, and at what level – global or local. Global inhibition suggests that the whole lexicon of a language is inhibited, whereas local inhibition refers to the inhibition of a small group of semantically and/or phonologically related items, or even the inhibition of a single item in the non-target language (Colzato et al., 2008; see Green, 2011, for an overview of different types of inhibition).

One way to investigate which of the two possibilities, IC or non-IC, is most accurate in predicting naming latencies, is to make use of long-lag morphological priming experiments. In long-lag priming experiments, the prime and target are separated from each other by several intervening items. IC models assuming global inhibition make a specific prediction for these types of experiments. If a language switch causes inhibition of the non-target language, then it is expected that any heightened activation of the prime and its related items would be inhibited after a language switch has occurred. That in turn would result in a reduced facilitation if not inhibition of the target item. For instance, when a prime in language A is followed by a language switch to language B, then the heightened activation of this prime and at least its related items will be decreased by the inhibition exerted by language B. Therefore, priming of a target in language A would possibly not be measurable anymore after this language switch.

Long-lag morphological priming was first used by Zwitserlood et al. (2000), who showed that morphological priming, a form of priming where the prime is morphologically related to the target, survives intervening lags of ten trials. However, effects of semantic and phonological priming were not obtainable anymore at those lags. These findings suggest that priming in those cases does not occur at a phonological or semantic level, but takes place at a separate morphological level. Koester and Schiller (2008, 2011) replicated these results in a combined behavioral and ERP study, and later in an fMRI study, showing that Dutch compounds prime morphologically related target picture names. Mere form overlap, as in *jasmijn* ‘jasmine’ – JAS ‘coat’ did not facilitate picture naming, suggesting that morphological priming is indeed another form of priming, different from identity priming (Wheeldon and Monsell, 1992). Their ERP data showed a reduced N400 component in posterior scalp regions when targets were preceded by morphologically related primes, but not when items with mere form overlap preceded them. Generally, an increased N400 component can be measured when participants are presented with unexpected items (Kutas and Federmeier, 2011). As the priming of a target item subconsciously prepares a participant for what is coming next, a primed target is less unexpected compared to an unprimed target, and therefore a reduced N400 peak is expected. The Koester and Schiller (2011) study found a neural priming effect in the left inferior frontal gyrus.

Interestingly, both transparent primes, where the target word is semantically related to one of the constituents of the compound prime (e.g., *eksternest* ‘magpie nest’ – EKSTER ‘magpie’), and opaque primes, where there is no semantic relationship between the compound and the target (e.g., *eksteroog* ‘corn,’ lit. ‘magpie eye’ – EKSTER ‘magpie’), resulted in faster naming latencies. These results suggest that complex words that need to be stored as wholes due to their non-decompositional semantics, the opaque compounds, are not only represented as wholes in the lexicon, but are also parsed into their constituents. This is in line with Baayen et al.’s (1997) parallel dual-route model, where both a computational and a storage component work in parallel when producing or retrieving a complex word.

However it seems that L2 speakers rely much more on a storage component than on computational processes (Brovetto and Ullman, 2001; Ullman, 2005; Silva and Clahsen, 2008). Brovetto and Ullman (2001) report on a speeded production task where high-frequency irregular past tense verbs were responded to faster, but high-frequency regular past tense forms did not show any frequency effects in L1 speakers. They argue that this reflects processes of storage for the irregulars, but computational processes for the regular forms. L2 speakers, however, showed frequency effects for both the irregular forms and for the regulars, indicating that their L2 knowledge relied much more on storage (but see Baayen et al. (2002) for a more subtle view on the balance of storage and computation in L1 speakers). Ullman (2005) proposes in his declarative/procedural model of memory that L2 speakers rely more on the declarative memory system and less on the procedural system – in other words, instead of parsing morphological complex forms, L2 speakers are expected to employ full-form storage much more than L1 speakers. Silva and Clahsen (2008) conducted several masked priming experiments in which they also found that L2 speakers process morphologically complex words differently from L1 speakers, relying much more on full-form storage than on computational processes. In their L1 groups, clear priming effects for complex words were found, where the prime consisted of an inflected or derived word form, and its simplex form as the target. In their group of L2 speakers, consisting of Chinese, Japanese and German learners of English, only identity priming was found, but no priming for the morphologically related forms.

Hahne et al. (2006) report on a behavioral and ERP study where they, however, did find evidence for both processes of storage and computation in L2 speakers. Their participants responded differently to violations of regular and irregular inflections – the first elicited an anterior negativity and a P600, whereas the latter resulted in an N400 effect. Their ERPs were very similar to those of L1 speakers.

Morphologically complex words, both transparent and opaque compounds, also facilitate naming their constituents when participants have to switch between their native language and their L2. Verdonschot et al. (2012) conducted a long-lag morphological priming experiment where participants had to switch between Dutch and English. They instructed Dutch (L1) – English (L2) bilinguals to read aloud words and to name pictures switching between Dutch and English. The primes and targets were presented in Dutch, whereas the intervening trials were presented in English for the switch blocks, and in Dutch during non-switch blocks. The primes consisted of Dutch compound words from which one of the constituents was identical to the target picture name. They used both transparent primes (e.g., *jaszak* ‘coat pocket’) and opaque primes (e.g., *grapjas* ‘funny person,’ lit. ‘joke coat’). Results showed that targets combined with morphologically related compounds, both transparent and opaque, yielded significantly faster naming latencies than targets preceded by morphologically unrelated primes. Despite the intervening language switch, priming effects still occurred, which according to the authors suggested that in switching to L2, no reactive inhibition is employed to suppress activation at the morphological level in L1 – for otherwise the heightened activation levels of the L1 primes would likely not have survived the repeated activation of the L2 and supposed inhibition of L1, and could therefore not have facilitated target picture naming.

However, it is possible that the dominant L1 (i.e., Dutch) is represented much stronger in the brain than the L2 (English) in Dutch-English bilinguals. If this were the case, then these inhibitory effects might be minor compared to the strength of the Dutch representation. This could mean that even though inhibition has taken place, priming effects are still measurable as the L2 was not strong enough to suppress all activation of the L1. In other words, the findings from Verdonschot et al. (2012) are still compatible with an inhibitory model where switching to another language causes the former language to be reactively inhibited by the language currently in use. Moreover, as the experiment was conducted in Dutch, in a Dutch-speaking country, with Dutch L1 speakers who would use Dutch in the majority of their daily lives, it is not unlikely that their Dutch lexical representations were much stronger activated overall. Therefore, if inhibition indeed plays a role, then one would expect that priming effects should be absent when the two languages are switched (e.g., using English primes/targets and Dutch intervening trials). If indeed Dutch is represented much stronger, and if there are mechanisms of inhibition employed when switching between languages, then the effect of inhibition caused by the Dutch intervening trials should be much larger than the priming effect of the English primes and targets, and therefore it is very likely that priming effects do not appear anymore or are at least so much reduced that they cannot be measured anymore by current experimental methods.

Given the previous research, several questions emerged. First of all, we aimed to replicate the morphological priming effects after a language switch, but then using L1 intervening items and L2 primes and targets, for reasons explained above. As a consequence, the experiment would involve priming in an L2. Therefore, not only an experiment with a language switch was needed, but also an experiment completely conducted in L2, to see whether morphological priming would occur at all in an L2. As L1 speakers seem to parse both transparent and opaque compounds (Koester and Schiller, 2008, 2011; Verdonschot et al., 2012), the question arises whether L2 speakers might process these two types of compounds differently. If they parse opaque compounds, like native speakers do, a morphological priming effect for opaque compounds is expected. If, however, they would simply retrieve the stored full forms, no morphological priming of the opaque forms would be observable. All these questions can be addressed by using a similar experimental design as in Verdonschot et al. (2012) but with the primes and targets in L2, English is this case, and the fillers in L1, Dutch.

Furthermore, we tested whether we would observe an N400 effect in morphological priming in an L2. Reduced N400 peaks have been found to occur in lexical priming paradigms (Kutas and Federmeier, 2011). Moreover, several studies have found N400 effects in early L2 speakers in semantic, associative, and categorical priming paradigms (Mueller, 2005). Therefore, it is expected that, if indeed there is morphological priming occurring in L2, there will be a reduced N400 effect in both the transparent and opaque conditions, both in the non-switch (only English items) and in the switch block. This was indeed observed in the ERP study of Koester and Schiller (2008) for morphological priming in the L1. When measured in late L2 learners (acquired their L2 after the age of 11), the N400 peak is often delayed and has a decreased amplitude (Mueller, 2005). However, as most of our participants have acquired English already in primary school, starting with classes at age 10, we do not expect them to behave very differently from native speakers.

We were able to bring all these elements together in the following experiment. We had Dutch speakers read aloud words and name pictures in a long-lag priming experiment, consisting of an English block and a block where they had to switch between English and Dutch. We collected both behavioral and ERP data in order to get a more fine-grained idea of the underlying processes of morphological processing in bilinguals.

## MATERIALS AND METHODS

### PARTICIPANTS

Thirty-six Dutch-English bilingual speakers currently enrolled in higher education or with a graduate degree in higher education (18 female, average 24.2 years), who had not participated in the Verdonschot et al. (2012) study, participated in the experiment. All had normal or corrected-to-normal visual and auditory acuity. They completed a questionnaire, which included general and language-specific questions. They were asked to rate their Dutch and English proficiency on a scale from 1–10 (with 1: very poor and 10: native-like). The average self-assessment of English proficiency was 7.8 (SD = 1.2) and Dutch proficiency 9.8 (SD = 0.5). Participants were also asked about their average proportion of English use per day. On average their percentage of English use per day, with respect to their use of Dutch, was 21.1% for speaking, 52.8% for reading, and 47.1% for listening. All participants gave informed consent and took part in an off-line English proficiency assessment (Meara and Buxton, 1987) ^1^.

Four participants were excluded from the EEG analysis due to excessive movement artifacts consisting of eye blinks and/or muscular activity, and three were not included because they were left-handed. The remaining 29 were on average 23.6 years of age (15 male).

### STIMULUS MATERIAL

The target stimulus set consisted of 36 black-and-white line drawings of concrete objects. Each target picture was combined with three different English compound words as primes. Two of the primes were morphologically related to the target, as one of their constituents was identical to the target picture name. The third type of prime was used as a control and therefore was neither morphologically, phonologically, nor semantically related to the target. The two morphologically related primes were either transparent, with the compound semantically related to the picture name, or opaque, where the compound is not semantically related to the picture name. An example of a transparent prime-target combination is moonlight-MOON. The first constituent of the compound is identical to the target MOON, and both the constituent and the target are identical in meaning. The opaque variant used in the experiment is honeymoon-MOON. Here, the constituent ‘moon’ of ‘honeymoon’ does not literally mean ‘moon’ in the compound it appears in. The compound ‘earring’ was used as the unrelated prime to the target MOON. It is neither phonologically nor semantically related to the word ‘moon.’

Word frequency, number of syllables, word length in phonemes, word length in letters, and stress position were matched – see **Table 1** for more information. Zwitserlood et al. (2000) have shown that the position of the target morpheme does not influence morphological priming – both the first and second constituent of compounds cause faster naming latencies in morphologically related targets. This finding has also been replicated in Koester and Schiller (2008) and Verdonschot et al. (2012). Thus, the position of the target morpheme was evenly distributed across conditions, in half of the cases as the first constituent, and in the other half as the second constituent. Also, only compounds written as one orthographic word were used, as De Jong et al. (2002) have found that compounds written with a space between the constituents are processed differently from compounds that are written as one orthographic word.

**Table 1:** Mean and SD (between parentheses) of the number of syllables, word frequency per million, number of phonemes, word length and stress position for each prime type and for the targets.

Prime type	# of syllables	Word frequency	(per million)	# of phonemes	Word length	Stress position
Opaque	2.39 (0.6)	6.44 (17.2)	7.61 (1.3)	8.56 (1.3)	1 (0)
Transparent	2.22 (0.4)	3.17 (5.2)	7.53 (1.3)	8.42 (1.2)	1 (0)
Unrelated	2.22 (0.4)	5.39 (10.8)	7.39 (1.1)	8.47 (1.3)	1 (0)
Target	1.14 (0.4)	154.47 (170.9)	3.78 (0.9)	4.17 (0.9)	1 (0)

To assess the semantic transparency of the opaque and transparent primes, a group of 31 students who did not participate in the experiment was asked to rate the semantic relationship of each target picture name to a constituent in either a transparent or opaque prime. They rated this relationship on a seven-point scale (1: not at all semantically related, 7: identical in meaning). Transparent compounds were rated more semantically related (5.9) than opaque compounds (2.9), *t*(70) = 14.6; *p* < 0.01. For all 36 targets, the transparent primes received on average higher scores than the opaque primes.

As a long-lag morphological priming design was employed, additional fillers to create intervening trials were used. As it was crucial that participants actually accessed their L1 in the switch block, we also employed pictures as intervening items. By using pictures, participants could not just rely on an orthography-to-speech route where they would not have to access the concepts themselves, and thereby possibly not the indicated language. Therefore, these intervening trials consisted of both words and pictures. An additional 25 pictures and 140 English and 140 Dutch filler words were selected.

In the appendix, an overview of all prime-target combinations used in the experiment can be found.

### DESIGN

For this experiment, the design was identical to Verdonschot et al. (2012). The experiment was designed and controlled using E-prime 2.0 (Psychology Software Tools). A 2 (Block Type: Switch vs. non-Switch) × 3 (Prime Type: Opaque, Transparent, and Unrelated) design was implemented, using six different experimental lists. The lists consisted of two different orders and three different prime-target combinations. Each participant saw each picture only twice – once in the non-switch condition, and once in the switch condition. This resulted in 72 (2 × 36) target trials per participant over all blocks. This way, participants did not see a target twice in the same condition and all targets were tested in all conditions across all participants. In **Table 2**, an example target with its three prime conditions is given.

**Table 2:** Example of a target with all three prime types – transparent, opaque and unrelated.

Prime type	Example (Prime)	Example (Target)
Transparent	moonlight	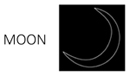
Opaque	honeymoon	
Unrelated	earring	

**FIGURE 1:**
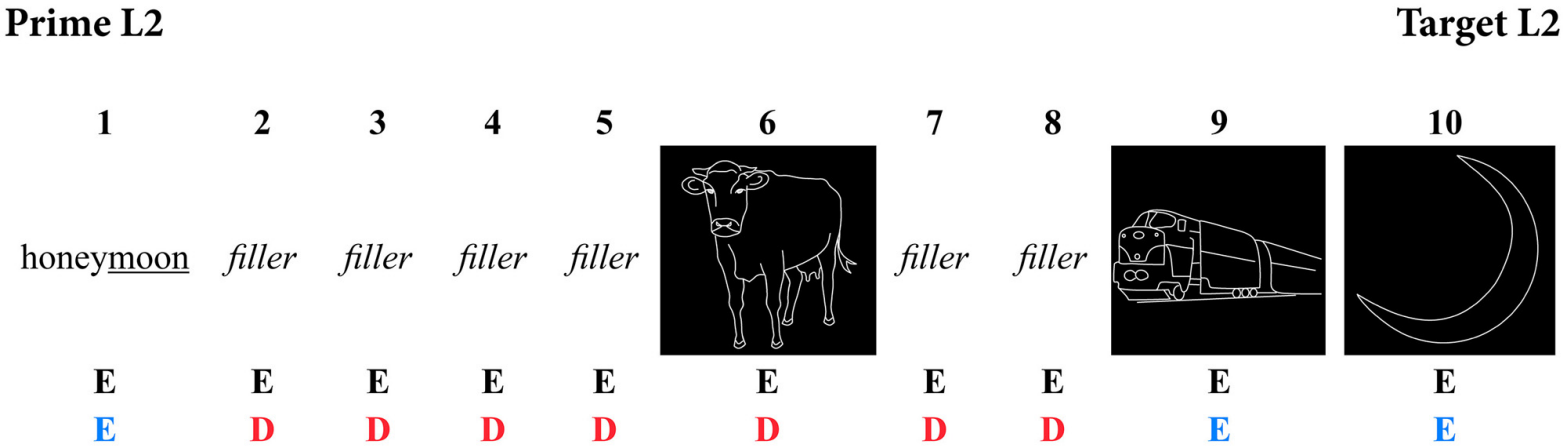
**Example sequence of a prime-target combination in both non-switch and switch blocks (E, English trial; D, Dutch trial)**.

Between each prime and target, filler items were included to create intervening trials. Previous experiments have shown that morphological priming effects even survive lags of up to 10 items (Zwitserlood et al., 2000), but to reduce the length of the experiment, only lags of either seven or eight items long were used. Each trial consisted of both pictures and words. They were positioned in the experiment such that intervening trials did not contain any items that were phonologically or semantically related to the following target picture in either language. Before every target picture, another picture was inserted that was to be named in English, to prepare participants to naming pictures instead of reading words, and in the switch blocks, also to avoid any additional language switching costs. These pre-stimulus pictures were also neither phonologically nor semantically related to the target pictures. See **Figure 1** for a prime-target example in both a non-switch and a switch block. To avoid any order expectation, additional sequences of words and pictures that did not match the order of a regular prime-target sequence were included.

### PROCEDURE

Before the start of the experiment, participants were given information about the experiment (written in Dutch), completed a questionnaire about general and language-specific information, and gave written informed consent. Participants were given 5 m to familiarize themselves with the Dutch and English names of the pictures used in the experiment by studying a booklet. In the booklet, all pictures were printed accompanied by their Dutch and English name, the Dutch name printed in a red font, and the English name printed in a blue font. Next, participants were seated individually in front of a computer screen in a quiet room, and were connected to an EEG setup. On the computer screen, Dutch instructions were presented in white letters against a black background. The participants were asked to read aloud words and name pictures as fast and accurately as possible. A voice-key (SR-BOX) was used to measure the naming latencies.

First, a practice block was administered which was identical in form to a switch block. This block consisted of 50 words and pictures; participants had to repeatedly switch between reading words and naming pictures in both Dutch and English. This way, the participants could familiarize themselves with the task, and the experimenter was able to see whether the voice-key was reacting appropriately to the voice of the participant.

The main experiment consisted of two blocks, one of which only contained English words and English pictures (the non-switch block), and the other which contained English primes and targets and Dutch intervening trials (the switch block). In the non-switch block, all words and pictures were presented in white against a black background. In the switch block, red words and a red frame indicated that the participants had to use Dutch, whereas blue indicated that English was to be used. The words were already written in the target language, and no translation was required – the colors were added to facilitate picture naming in the correct language.

Each trial began with a fixation cross in the middle of the screen for 250 ms, followed by a blank screen for 250 ms. Next, a picture or word was presented for 400 ms, after which it disappeared from the screen and participants had an additional 1,100 ms to name the item. The experimenter assessed the validity of the trial on-line, indicating whether word errors or voice-key errors occurred. After each experiment, participants completed an off-line English proficiency assessment task (Meara and Buxton, 1987).

### EEG RECORDINGS

The EEG was recorded using 32 Ag/AgCl electrodes (BioSemi ActiveTwo), which were placed on the scalp sites according to the standards of the American Electroencephalographic Society (1991). Eye blinks were measured by two flat electrodes placed at the sub- and supra-orbital ridge of the left eye (VEOG1 and VEOG2), horizontal eye movements were measured by two flat electrodes placed at the right and left outer canthi (HEOG1 and HEOG2), and two flat electrodes were placed at the two mastoids. The electrodes CMS and DRL were used as ground references. The EEG signal was later re-referenced off-line using the mean of the two mastoids. Sampling occurred at 512 Hz, and a band-pass filter of 0.01–30 Hz was applied off-line.

### DATA ANALYSIS

Participant errors (7.7%) and voice key errors (8.3%) were excluded from further analysis. Reaction times that deviated more than 2.5 SDs from a participant’s mean per condition (4.1%) were removed ^2^. The trimmed data were non-normally distributed as indicated by a Shapiro–Wilk test (all *p*s < 0.05); therefore, it was decided to take the natural log of the reaction times. A repeated-measures ANOVA on both the trimmed data and on the trimmed, log-transformed data was performed. ANOVAs from both sets are reported.

Mauchley’s test showed violations of sphericity against the factor Prime Type (*F*1) and the interaction of Prime Type and Block (both *F*1 and *F*2), *W*(2) = 0.73, *p* < 0.01, *W*(2) = 0.70, *p* < 0.01, and *W*(2) = 0.70, *p* < 0.01, respectively. A 2 × 3 repeated-measures ANOVA was conducted with a Greenhouse-Geisser correction (ε = 0.78, ε = 0.79, ε = 0.77) to test for statistical significance, with Block and Prime Type as factors, and participants (*F*1) and stimuli (*F*2) as random factors. For all six different experimental conditions, the mean, SD, and 95% confidence intervals were calculated for picture naming latencies.

Regarding the ERP data, four participants were excluded from the analysis due to excessive movement artifacts. Trials were mostly excluded because of movement artifacts due to eye blinks and overt speech. These were trials with amplitudes below –200 μV, above 200 μV or trials within which there was an absolute voltage difference of more than 200 μV. Also, all trials that were responded to incorrectly by participants were excluded from the EEG analysis. Therefore, a total of 41.3% of all trials was used in the averaging procedure (41.1% for the opaque non-switch condition, 40.6% for the transparent non-switch condition, 38.3% for the unrelated non-switch condition, 43.8, 40.6, and 43.5% for the opaque, transparent, and unrelated switch condition, respectively).

Mean amplitude ERPs were calculated for each participant separately, using a time window of 100 ms prior and 600 ms following picture onset. Between 0 and 600 ms post stimulus onset, mean amplitudes per time windows of 50 ms, with an overlap of 25 ms, were evaluated for an N400. Repeated-measures ANOVAs with Greenhouse-Geisser corrections were used to analyze the ERP amplitudes.

## RESULTS

### BEHAVIORAL DATA

There is a main effect of Block, *F_1_*(1, 35) = 80.55, *MSE* = 5433.07, *p* < 0.01, η^2^= 0.18; *F_2_*(1, 35) = 79.60, *MSE* = 5598.64, *p* < 0.01, η^2^ = 0.20; *min F′*(1,70) = 40.04, *p* < 0.01, showing that there is a significant difference in naming latencies between trials from the switch block and from the non-switch block. After the log-transformation results were similar, again showing a main effect of Block: *F_1_*(1,35) = 76.02, *MSE* = 0.03, *p* < 0.01, η^2^ = 0.16; *F_2_*(1,35) = 92.90, *MSE* = 0.03, *p* < 0.01, η^2^ = 0.20; *min F′*(1,70) = 41.8, *p* < 0.01. Participants took on average 85 ms longer to name items when they had to constantly switch between Dutch and English. The Block Type did not affect accuracy; participants made on average 2.6% errors in the switch blocks, compared to 2.9% in the non-switch blocks. There was also a main effect of Prime Type, *F*_1_(2,70) = 21.64, *MSE* = 2926.42, *p* < 0.01, η^2^ = 0.06; *F_2_*(2,70) = 19.11, *MSE* = 2804.29, *p* < 0.01, η^2^ = 0.06; *min F′*(2,140) = 10.15, *p* < 0.01, indicating that the type of prime influenced response latencies. After the log-transformation of the per-item scores, the statistics showed similar values: *F*_1_(2,70) = 20.95, *MSE* = 0.02, *p* < 0.01, η^2^ = 0.05, *F*_2_(2,70) = 19.17, *MSE* = 0.02, *p* < 0.01, η^2^ = 0.06; *min F′*(2,140) = 10.01, *p* < 0.01. The interactions between Block and Prime Type did not reach significance, *F*_1_(2,70) = 3.08, *MSE* = 2789.46, *p* = 0.052, η^2^ = 0.009; *F*_2_(2,70) = 1.34, *MSE* = 2858.76, *p* = 0.27, η^2^ = 0.004; *min F′*(2,121) = 0.93, *p* = 0.40. After the log transform, the interaction was not significant either: *F*_1_(2,70) = 1.60, *MSE* = 0.02, *p* = 0.21, η^2^ = 0.004; *F*_2_(2,70) = 1.34, *MSE* = 0.02, *p* = 0.48, η^2^ = 0.002; *min F′*(2,139) = 0.73, *p* = 0.48.

**FIGURE 2:**
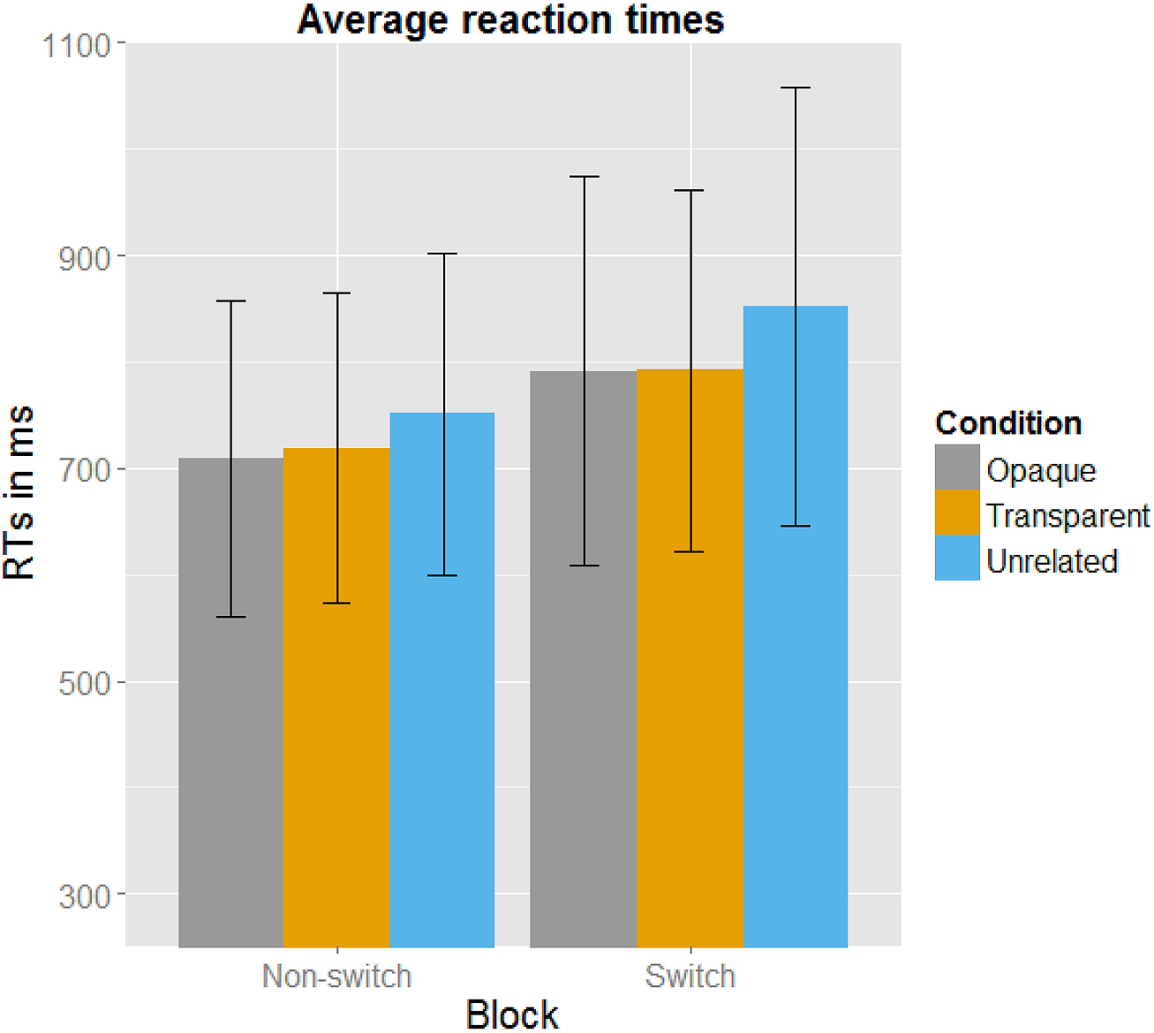
**Averaged reaction times in milliseconds per Condition per Block, with error bars indicating the SD.** It can be clearly seen that participants in the Switch Block were overall slower. Also, the Unrelated Condition has slower reaction latencies than the Opaque and Transparent Condition.

*Post hoc* paired *t-*tests revealed that in the non-switch block, targets primed by both opaque and transparent primes differed significantly from the control targets. As expected, there was no significant difference between the opaque and the transparent condition. In the switch block, both opaque and transparent primes resulted in significant faster naming latencies of the target picture than the control (unrelated) primes did. There was again no significant difference between transparent and opaque primes. See **Figure 2** for a plot with the average reaction times. All mean reaction times, SD, 95% confidence intervals, error rates, and the *post hoc* paired *t*-tests are shown in **Table 3**.

**Table 3:** Overview of all mean reactions times (RT), error rates (E), 95% confidence intervals, differences between the conditions, and paired comparisons.

	Non-switch				Switch			
	*RT*	% *E*	*CI*		*RT*	*% E*	*CI*	
Opaque	709 (148)	2.0	694, 724		791 (183)	2.1	771, 810	
Transparent	719 (146)	2.5	703, 734		792 (170)	1.9	774, 810	
Unrelated	751 (151)	2.4	735, 767		851 (206)	2.8	829, 874	

	Δ ***RT***	Δ ***E***	***t**_**1**_* **(35)**	***t**_**2**_* **(35)**	Δ ***RT***	Δ ***E***	***t**_**1**_* **(35)**	***t**_**2**_* **(35)**

O-U	42	0.4	-3.6, *p* = .001	-3.7, *p* = 0.001	60	0.7	-4.5, *p* < 0.001	-2.9, *p* = 0.006
T-U	32	0.1	-2.1, *p* = 0.045	-2.7, *p* = 0.010	59	0.9	-4.2, *p* < 0.001	-5.1, *p* < 0.001
O-T	10	0.5	-1.2, *p* = 0.243	-1.3, *p* = 0.195	1	0.2	-0.1, *p* = 0.948	0.87, *p* = 0.389

*Standard deviations are between parentheses*.

### ERP DATA

**FIGURE 3:**
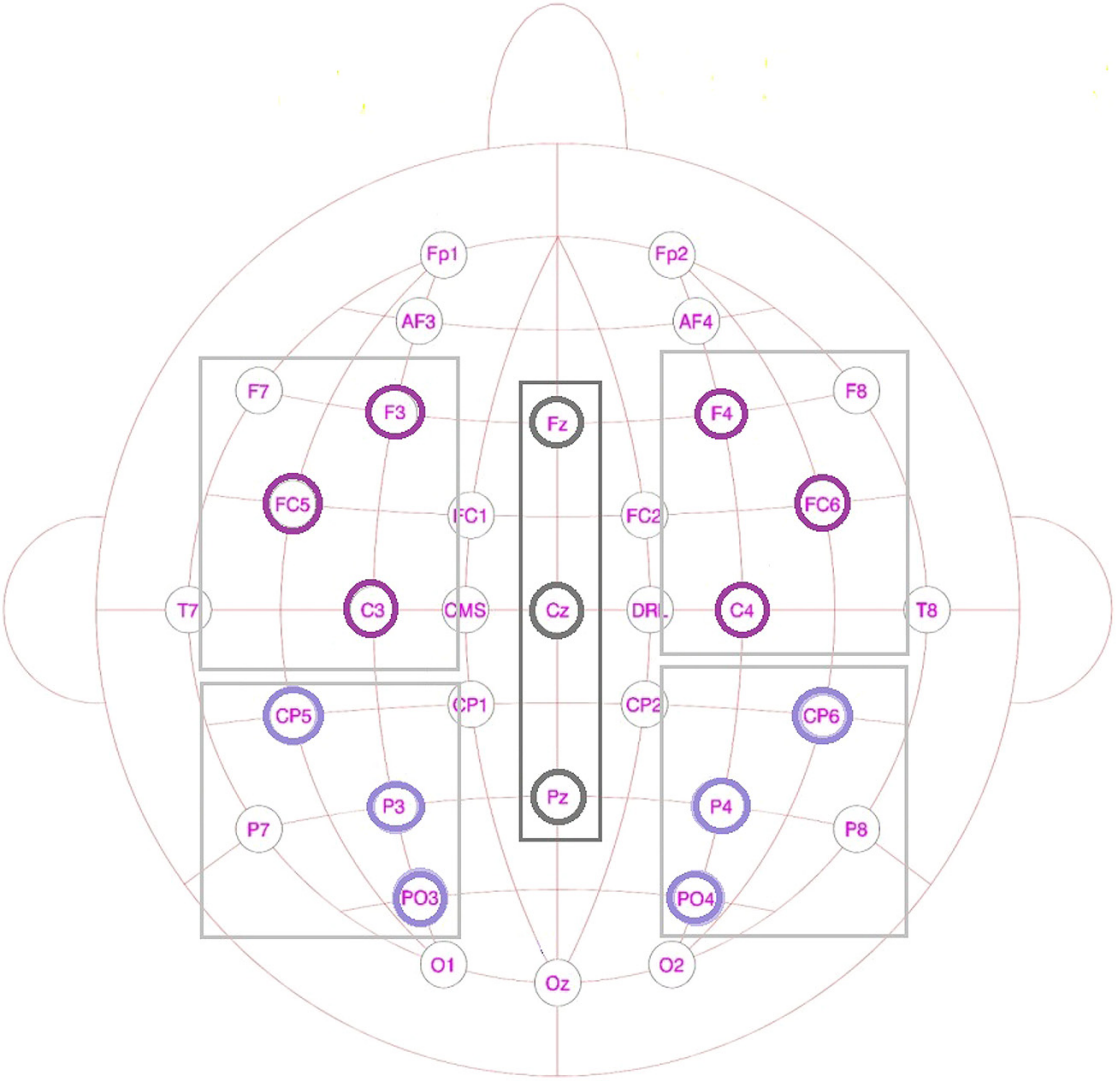
**Schematic drawing of the scalp showing the lateral regions and the midline electrodes used in the EEG analysis**.

One set of analyses was conducted for lateral sites, divided into four different Regions of Interest (ROIs; see **Figure 3**): anterior-left (F3, FC5, C3), anterior-right (F4, FC6, C4), posterior-left (CP5, P3, PO3), and posterior-right (CP6, P4, PO4), with the factors Prime Type (3), Block (2), and ROI (4) as factor. Another set comprised the midline electrodes Fz, Cz, and Pz, with a Prime Type by Block by Electrode (3) design. All the mean amplitude values were compared in a repeated-measures ANOVA per blocks of 50 ms, with an overlap of 25 ms each, within the 0 and 600 ms post-stimulus-onset time window. The time window between 400 and 575 ms showed a significant interaction or trends toward an interaction between Block and Condition for both the midline electrodes and the lateral regions.

**FIGURE 4:**
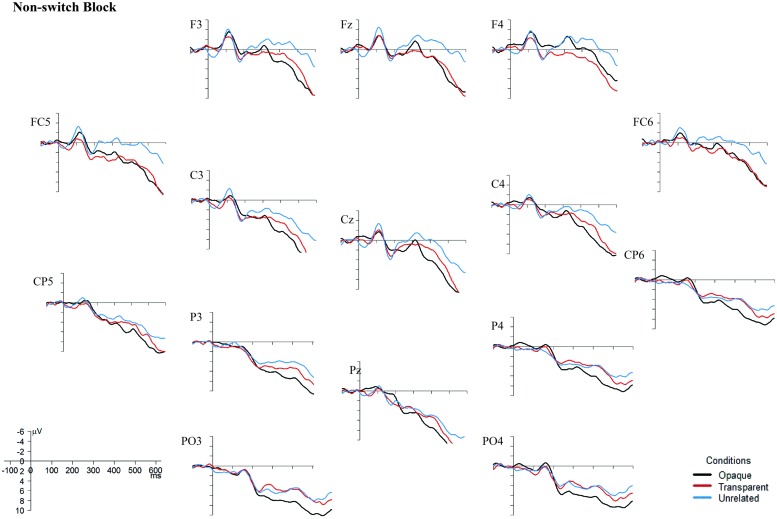
**Grand Averages ERPs, superimposed for the opaque, transparent, and unrelated conditions in the non-switch block.** The ERPs are time-locked to the onset of the target picture, and a 10 Hz low-pass filter was applied to smoothen the graphs. Negativity is plotted upward. Images were created with the statistical software R (R Core Team, 2014).

**FIGURE 5:**
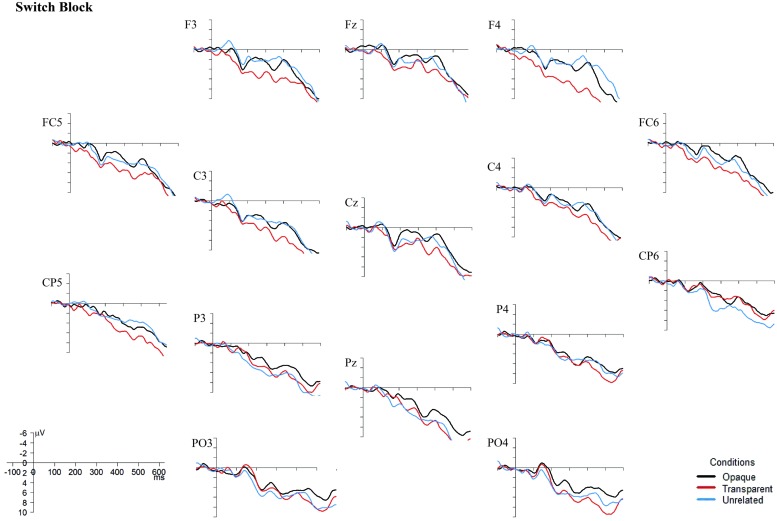
**Grand Averages ERPs, superimposed for the opaque, transparent, and unrelated conditions in the switch block.** The ERPs are time-locked to the onset of the target picture, and a 10 Hz low-pass filter was applied to smoothen the graphs. Negativity is plotted upward. Images were created with the statistical software R (R Core Team, 2014).

Although the behavioral data showed no difference between the non-switch and the switch block, the EEG data do (see **Figures 4** and **5**). In the non-switch block, the unrelated condition indicates an increased negativity around 400 ms post-stimulus onset in frontal regions, whereas in the switch block the transparent condition is indicating a reduced N400. As the graphs seem to show a different pattern for the non-switch and the switch block, and the factor Block was involved in significant interactions in both the lateral and midline regions, it was decided to perform separate analyses for the 400–575 ms time window for the non-switch and the switch block. For the midline electrodes, there was no significant interaction between Condition and Electrode in neither the non-switch nor the switch block, *F*(4,88) = 0.92, *p* = 0.42, and *F*(4,88) = 0.76, *p* = 0.50. Condition is a significant main effect only in the non-switch block, *F*(2,44) = 4.81, *p* = 0.01. *Post hoc* Tukey tests indicate that there is no difference between the opaque and transparent condition (*p* = 0.79), but that the unrelated condition is significantly different from the opaque condition (*p* = 0.01) and near-significant from the transparent condition (*p* = 0.08).

Since there was also a near-significant interaction between Block and Condition (*F*(2,44) = 3.47, *p* = 0.06) in the lateral regions, separate analyses for the non-switch and switch block were conducted. In the switch block, there is no significant interaction between Condition and ROI, *F*(6,132) = 0.47, *p* = 0.64, and no main effects of neither Condition nor ROI, *F*(2,44) = 4.42, *p* = 0.25, and *F*(3,66) = 0.42, *p* = 0.63. In the non-switch condition, there is a significant main effect of Condition, *F*(2,44) = 3.90, *p* = 0.03, and a main effect of ROI, *F*(3,66) = 5.68, *p* = 0.002. *Post hoc* Tukey tests for the factor Condition in the non-switch block show no difference between the opaque and the transparent condition (*p* = 0.32), but a significant difference between the unrelated condition and both the transparent (*p* < 0.01) and opaque condition (*p* < 0.01).

## DISCUSSION

Participants took on average longer to name items in the switch block than in the non-switch block. These switching costs were expected, as the task in the switch blocks was more difficult. Participants not only had to switch between reading words and naming pictures, but were also required to switch between languages (Koch et al., 2010; Verdonschot et al., 2012). In both the non-switch and the switch blocks, the participants named targets preceded by morphologically related primes significantly faster than target pictures preceded by unrelated primes. Whether the primes were opaque or transparent compounds, did not have any influence on priming effects, as both types resulted in statistically faster naming latencies and there were no significant differences between those two conditions. These results lend further support to models of language production where morphemes constitute a separate level, which is independent of semantics (e.g., Levelt et al., 1999). What is even more interesting is that this study clearly suggests that this independent morpheme level also has to be present in the L2 of proficient bilinguals.

The independence of morphology from semantics is further supported by the fact that in this and other studies (Koester and Schiller, 2008, 2011; Verdonschot et al., 2012) there is no statistical difference between the effect of both transparent and opaque primes. Transparent and opaque compounds differ from each other on whether their meaning is compositional, so that you can derive the meaning of the whole compound from the meaning of its constituents, as in ‘moonlight,’ or whether the compound is not compositional, so that the meaning of the whole compound is not derivable from the meanings of its constituents, as in ‘honeymoon.’ Thus, in transparent compounds the constituent identical to the target still shares semantic content, whereas in opaque compounds this is not the case. The shared morpheme of the prime and target only shares its form, but not its meaning. However, as Koester and Schiller have shown, form overlap does not lead to priming in long-lag designs. Therefore, in order to account for the presence of priming effects in the opaque condition, at least some form of processing of separate morphemes of complex, opaque words has to be assumed. Consequently, even though the meaning of opaque compounds such as ‘butterfly’ needs to be stored, the separate morphemes of the compound are also available. This seems to be the case for opaque compounds both in the L1 (Verdonschot et al., 2012), but also in an L2, as this study has shown.

Importantly, the results of this study suggest that morphological priming does occur in L2 in proficient bilinguals. Considering only the behavioral data, it also seems to be the case that a language switch to L1 between the prime and target does not interfere with priming effects, suggesting that a language switch from L2 to L1 does not result in reactive inhibition of, at least, morphologically related items in L2. However, the ERP data seem to suggest otherwise. In the non-switch block, a reduced N400 effect was found for unrelated primes, corroborating with Koester and Schiller’s (2008) results. However, the ERP data from the switch block do not show any N400 effects. This might indicate different participant strategies for the non-switch and the switch block.

The language switch might have made the relation between prime and target too salient so that participants recognized this relation. Therefore, they could have employed a post-lexical checking strategy that facilitated naming of the target items, which also resulted in faster response latencies. After having uttered the prime item, hypotheses about possible following items could have been checked against the concept accessed when naming the target picture. This could then have sped up the naming process, or it might have even sped up the recognition of the picture itself or the access of the concept related to the picture. Thus, whereas the patterns seen in the non-switch block seem to reflect an automatic priming process, the patterns from the switch block could reflect a less automatic process where participants relied more on a post-lexical checking strategy. It is also possible that in the switch block, having to switch from one language to the other constantly has led to an increased activation of the translated concept in the other language. In that way, when a participant had to read out loud a compound in English, it might have led to increased activity not only of the English constituents of this compound, but also of the translated variants in Dutch. Previously, Christoffels et al. (2007) found a phonological activation for cognates in the non-response language. Therefore, it seems likely that also in this study participants might have activated the translated variants of cognate items, or even all translated variants in the non-response language, i.e., Dutch. These Dutch concepts might then later have facilitated picture naming in English. This process is different from a pure priming process in only English, but would also lead to faster picture naming. This could explain why the behavioral data show decreased reaction latencies in both the switch and the non-switch block, but why the ERP data only show an N400 effect in the non-switch block ^3^.

If it is indeed the case that participants used a different mechanism in the switch block, then it raises the question what this means for the conclusions that can be drawn for BLC. In any case, a full inhibition of the L2 could not have taken place, even if participants just relied on a post-lexical checking strategy. Otherwise they would not have been able to keep the concepts related to the primes active during the intervening Dutch trials. However, only items related to the prime could have been hold active until the target was encountered, which is compatible with an account assuming almost full inhibition of the non-target language, with just a very marginal activation of specific items. This is also compatible with an account assuming no inhibition at all.

The results of this priming study show that both transparent and opaque compounds in the L2 are parsed up to the morphological level, suggesting that even compounds that need to be stored as wholes, as their semantics are not compositional, are internally parsed. The results also indicate that behavioral data benefit from being augmented with EEG data, i.e., only the ERP data showed that participants were actually processing languages differently when switching between their L1 and their L2 from speaking only in their L2. Moreover, it has shown that accounts assuming full inhibition of the non-target language in bilinguals are not compatible with the observations made in Verdonschot et al. (2012) and the current study.

Combining ERP data with behavioral data in language switching paradigms, as well as using a diverse range of participants with different language backgrounds, language ecologies, and language proficiencies may shed further light on the issue of the representation of complex words.

## Conflict of Interest Statement

The authors declare that the research was conducted in the absence of any commercial or financial relationships that could be construed as a potential conflict of interest.

## Footnotes

1 Average scores were 4898/4414, with a SD of 130/353.

2 The errors and trimmed (removed) data were evenly distributed over the six conditions. The percentages are 2.9% for non-switch opaque, 3.0% for non-switch transparent, 3.2% for non-switch unrelated, 3.8% for switch opaque, 3.3% for switch transparent and 3.9% for the switch unrelated condition.

3 We thank an anonymous reviewer for this suggestion.
