# Supplementary material for: Morphological priming during language switching: an ERP study
Source: Front Hum Neurosci. 2014 Dec 12;8:995. doi: 10.3389/fnhum.2014.00995 (PMC4264473; doi:10.3389/fnhum.2014.00995)
Supplement: Supplementary file 1 [file Table_1.DOCX]

***Supplementary Material***

**Morphological priming during language switching: an ERP study**

**S.E. Lensink^1,2^*, R.G. Verdonschot^2,3^, N.O. Schiller^1,2^**

^1^ Leiden University Centre for Linguistics, Faculty of Humanities, Leiden University, Leiden, The Netherlands

^2^ Leiden Institute for Brain and Cognition, Leiden University, Leiden, The Netherlands

^3^ Graduate School of Languages and Cultures, Nagoya University, Nagoya, Japan

*** Correspondence:** Saskia E. Lensink, Leiden Institute for Brain and Cognition (LIBC) & Leiden University Centre for Linguistics (LUCL), Faculty of Humanities, Leiden University, Van Eyckhof 3, 2311 BV Leiden, The Netherlands.

E-mail: s.e.lensink@hum.leidenuniv.nl

1. **Stimulus set**

| **Target** | **Opaque prime** | **Transparent prime** | **Unrelated prime** |
| --- | --- | --- | --- |
| GOLD | goldfish | goldsmith | airplane |
| EGG | eggplant | eggcup | homework |
| WINE | winegum | wineglass | classmate |
| SUN | sunday | sunshine | toothpaste |
| WATER | watermelon | waterfall | pineapple |
| KEY* | keyword | keyhole | ashtray |
| SPEAR | spearmint | speargun | dishwasher |
| CROSS | crossword | crossroad | rainbow |
| BUTTER | butterfly | buttercream | locksmith |
| STONE | milestone | cobblestone | teardrop |
| BIRD* | ladybird | blackbird | postcard |
| TAIL* | cocktail | oxtail | bedroom |
| MILL | treadmill | windmill | backpack |
| HEAD | warhead | forehead | pancake |
| NAIL | thumbnail | fingernail | grandchild |
| BOX* | chatterbox | toolbox | sweetheart |
| WORM | bookworm | earthworm | seashore |
| COAT* | petticoat | raincoat | walnut |
| MOON | honeymoon | moonlight | earring |
| EYE | bullseye | eyelash | grapefruit |
| FLOWER* | cauliflower | flowerpot | chopstick |
| DOG* | underdog | dogfight | skyline |
| BOY* | cowboy | boyfriend | hourglass |
| GOAT | scapegoat | goatskin | popcorn |
| LACE* | shoelace | lacework | rattlesnake |
| HAND | shorthand | handshake | battleship |
| NECK | bottleneck | necktie | birthday |
| HORSE* | horseradish | racehorse | gunpowder |
| FIRE | firefly | campfire | pocketknife |
| STAR | starfish | polestar | backbone |
| CAT | catwalk | wildcat | bathtub |
| CHAIR* | chairman | wheelchair | sandcastle |
| TABLE | tablespoon | worktable | doorstep |
| GRASS | grasshopper | lemongrass | daydream |
| FOOT | footnote | barefoot | driveway |
| SHELL | shellshock | seashell | mousetrap |

Word marked with an * are non-cognates in Dutch and English
